# MSe Collision Energy Optimization for the Analysis of Membrane Proteins Using HDX-cIMS

**DOI:** 10.1021/jasms.4c00093

**Published:** 2024-06-06

**Authors:** Juan Pablo Rincon Pabon, Zulaikha Akbar, Argyris Politis

**Affiliations:** † Faculty of Biology, Medicine and Health, Division of Molecular and Cellular Function, The University of Manchester, Manchester M13 9PT, U.K.; ‡ Manchester Institute of Biotechnology, University of Manchester, Princess Street, Manchester M1 7DN, U.K.

## Abstract

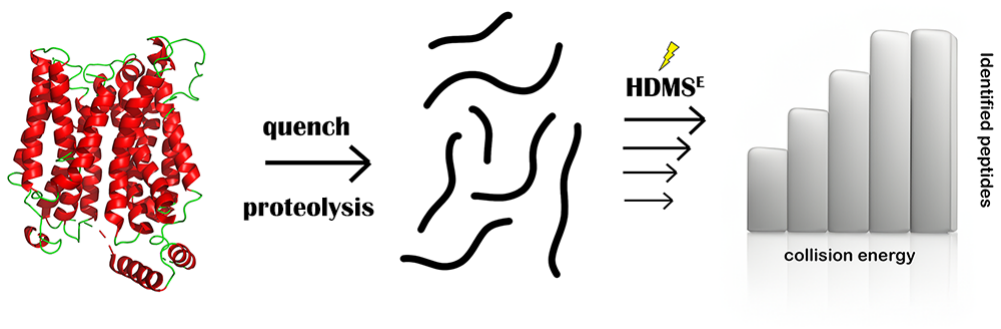

Hydrogen/deuterium exchange mass spectrometry (HDX-MS) has evolved as an essential technique in structural proteomics. The use of ion mobility separation (IMS) coupled to HDX-MS has increased the applicability of the technique to more complex systems and has been shown to improve data quality and robustness. The first step when running any HDX-MS workflow is to confirm the sequence and retention time of the peptides resulting from the proteolytic digestion of the nondeuterated protein. Here, we optimized the collision energy ramp of HDMS^E^ experiments for membrane proteins using a Waters SELECT SERIES cIMS-QTOF system following an HDX workflow using Phosphorylase B, XylE transporter, and Smoothened receptor (SMO) as model systems. Although collision energy (CE) ramp 10–50 eV gave the highest amount of positive identified peptides when using Phosphorylase B, XylE, and SMO, results suggest optimal CE ramps are protein specific, and different ramps can produce a unique set of peptides. We recommend cIMS users use different CE ramps in their HDMS^E^ experiments and pool the results to ensure maximum peptide identifications. The results show how selecting an appropriate CE ramp can change the sequence coverage of proteins ranging from 4 to 94%.

## Introduction

Hydrogen/deuterium exchange mass spectrometry (HDX-MS) has become an essential technique for probing protein conformation and dynamics over the past decade, owing to its ease of use, versatility, and increased robustness. Among the applications, HDX-MS has been used for epitope mapping,^1−3^ to probe conformational changes,^4−6^ to identify protein–protein interactions,^7,8^ to device protein mechanisms,^9^ or to characterize excipients,^10^ to name a few.

In a traditional HDX bottom-up experiment, proteins are incubated in a deuterated buffer for varying time points. This enables amide hydrogens to exchange with deuterated atoms present in the buffer. The hydrogen/deuterium rate of exchange depends on many factors but primarily on protein structure/hydrogen bonding.^11^ Afterwards, the reaction is quenched by decreasing the pH and temperature, and the protein is digested using nonspecific proteases such as Pepsin. Once peptides are obtained, they are desalted and separated by using reversed-phase chromatography to finally measure peptide masses by using mass spectrometry. Resulting changes in peptide masses provide information on structural changes and can be used to infer protein information. The underlying theory of HDX-MS and experimental recommendations are out of the scope of this paper and can be found elsewhere.^11−13^

As part of the traditional HDX-MS workflow, an initial peptide map using a nondeuterated sample must be obtained. The goal of this experiment is to both confirm the identity of the peptides following the proteolytic digestion and to stablish the retention time of each peptide.^14^ The use of different MS/MS fragmentation techniques is routinely used to confirm the sequence of the digested peptides. MS/MS fragmentation most commonly follows a data-dependent acquisition (DDA) mode, wherein precursor ions are selected for fragmentation based on their abundance and charge.^15^ While DDA is the most commonly used mode, reproducibility of precursor selection and long instrument cycle times make DDA applicability for HDX limited. To address DDA shortcomings, the data-independent acquisition (DIA) mode was introduced, wherein all ions within a small isolation window are fragmented despite their abundance or charge.^16^ This produces an unbiased but complex MS/MS spectra, that specialized software deconvolute to match the fragment ions to precursor ions.^17^

Several DIA methodologies have been introduced in the last two decades,^18^ such as MS^E^, wherein low and high collision energy (CE) scans are acquired one after the other, giving information on both precursor ions and fragment ions in a single run. Fragment ions are obtained using collision induced dissociation (CID), where precursor ions are accelerated under vacuum using high voltages and collided with an inert gas. Although MS^E^ spectra are complex, specialized software have made the technique mainstream and more recently, with the introduction of ion-mobility separation (IMS) to several commercial instruments, improvements to MS^E^ became available.^19^

IMS provides an orthogonal mode of separation by separating gas ions based on the interaction with an inert gas, making it possible to separate ions with the same chromatographic retention time. Owing to the numerous advantages, IMS has now been introduced to many omics workflows.^20^ Fundamental concepts, instrumentation, and applications of IMS are available elsewhere.^20^ The use of IMS in an HDX-MS workflow is ideal, increasing the peak capacity without increased analysis time and virtually no increase in back-exchange.^21^ HDX-IMS-MS has shown great results with improved sequence coverage for many different proteins^22,23^

The Synapt G2-Si mass spectrometer is a commercial instrument developed by Waters Corporation. This instrument includes a traveling wave IMS cell and has been largely commercialized and directed toward HDX users. IMS has been shown to improve the fragmentation efficiency by correlating the CE with the mobility of the ions.^16^ In IMS-MS^E^, also known as HDMS^E^, a fixed CE is applied to each individual IMS cycle, leading to the potential overfragmentation or underfragmentation of numerous peptides. To overcome this issue, ion-mobility dependent CE profiles (known as UDMS^E^) can be used and have shown to result in better fragmentation patterns and higher peptide identification rates.^16^

We previously developed a UDMS^E^ strategy specifically aimed to improve MS^E^ of peptic peptides in a HDX workflow using a Synapt G2-Si mass spectrometer.^24^ This strategy first runs a HDMS^E^ peptide map with a CE ramp of 25–45 eV; data is then processed using PLGS (Waters’ proprietary analysis software) and later passed through a Python script to calculate and modify the IM dependent CE of the peptides (coined as CE LUT), and later, a new peptide map experiment using the CE LUT profile is performed. We demonstrated that using our approach we could increase the sequence coverage in an HDX workflow using Phosphorylase B, AcrB, and an IgG2 compared to the traditional HDMS^E^ approach.^24^

On the other hand, the use of HDX-MS to probe membrane protein (MP) conformation and dynamics has been widely reported.^25−30^ Even though HDX-MS for MP is gaining popularity, it is still challenging due to the inherent hydrophobicity of the proteins, the complexity of the systems, and the need for membrane mimetics to solubilize the proteins.^31^ The presence of such membrane mimetics (e.g., detergents or lipids) can cause ion suppression, resulting in poor signal-to-noise ratio.^32^ To overcome possible sensitivity issues arising from poor digestion of the MP and to increase peak resolution, we introduced a state of the art SELECT SERIES cyclic ion mobility mass spectrometer (cIMS) developed by Waters Corporation^33^ into our HDX workflow. This instrument is based on a Synapt G2-Si mass spectrometer but uses a cyclic ion mobility device that improves the IM resolution and enables more complex experiments. Although MS^E^ using CE profiles associated with mobility data (UDMS^E^) of the peptides is theoretically possible with this instrument, the feature is not available yet, forcing the user to use a traditional CE ramp when running MS^E^ experiments.

Here, we optimized the CE ramp for HDMS^E^ of membrane protein peptic peptides following an HDX workflow in a Waters SELECT SERIES cIMS QTOF system. We employed Phosphorylase B, an MFS sugar transporter (XylE), and a G protein coupled receptor (SMO) as model systems.

## Experimental Section

### Materials

Phosphorylase B (PhosB) from rabbit muscle was purchased from Waters Corporation (Wilmslow, U.K.). *n*-Dodecyl-β-d-maltoside (DDM) was purchased from Avanti Polar Lipids (Alabaster, AL, USA). Optima grade 0.1% formic acid in water and 0.1% formic acid in acetonitrile blends were purchased from Fisher Scientific (Leicestershire, U.K.). Tris(2-carboxyethyl)phosphine hydrochloride (TCEP), guanidine hydrochloride, urea, sodium chloride (NaCl), 4-(2-hydroxyethyl)-1-piperazineethanesulfonic acid (HEPES), cholesteryl hemisuccinate (CHS), and sodium phosphate monobasic were purchased from Merck Life Science (Gillingham, U.K.). Smoothen receptor (SMO) was kindly provided by OMass therapeutics.

### XylE Expression and Purification

The detailed protocol to express and purify XylE has been published before.^29,34^ Briefly, the XylE WT was overexpressed in *E. coli* BL21-Gold (DE3) competent cells. Cells were transformed with a kanamycin-resistant pET28-a plasmid containing XylE-WT with a modified C-terminal 10x-histidine tag on Lysogeny Broth (LB) agar plates supplemented with kanamycin. A 100 mL starter culture was inoculated with a transformed colony and incubated overnight at 200 rpm at 37 °C. Overexpression was initiated by the transfer of 10 mL of starter culture to 1 L flasks containing LB-kanamycin. Bacteria was grown at 37 °C and 220 rpm until the OD_600_ value was 0.8. Expression was induced with 1 mM isopropy-β-d-1-thiogalactopyranoside (IPTG), and cells were harvested at 4000 rpm for 20 min. Pelleted cells were stored at −80 °C.

Thawed cells were resuspended in lysis buffer and passed through a cell disrupter at 25 kPsi and 4 °C before high-speed centrifugation at 12,000 rpm for 30 min. The resulting supernatant was harvested by ultracentrifugation at 38,000 rpm for 1 h. Pelleted membrane vesicles were resuspended in storage buffer and homogenized with a glass homogenizer before storage at −80 °C.

Membrane vesicles were solubilized for 2 h at 4 °C, and solubilized membrane proteins were isolated by ultracentrifugation at 38,000 rpm for 30 min. Supernatant was applied to TALON Metal Affinity Resin packed in TALON 2 mL Disposable Gravity Column pre-equilibrated with 96% SEC buffer. Resin was then washed four times with 85% SEC buffer and 15% elution buffer before elution. Eluted proteins were subjected to size exclusion chromatography with a Superdex 16/600 GL SEC column. Fractions containing XylE were collected and concentrated using Vivaspin concentrators (30 kDa cutoff). All samples were flash frozen and kept at −80 °C until use.

### LC-HDMS^E^ Method

Following an HDX workflow, all sample handling and dilution steps were performed using a dual head Trajan LEAP HDX automation system (Carrboro, NC, USA). For Phosphorylase B experiments, 2.5 μL of a 20 μM stock solution was diluted with 100 μL of equilibration buffer (10 mM sodium phosphate, pH: 7.5). Then, 100 μL of the protein solution was quenched with 100 μL of precooled quench buffer (100 mM sodium phosphate, pH: 2.3). Immediately after, 195 μL of the sample was injected into a chromatography cabinet connected to two ACQUITY I Class binary pumps. Sample was passed at 200 μL min^–1^ with 0.1% formic acid in water through a pepsin column (2.1 × 30 mm) immobilized in house kept at 20 °C for 210 s. Resulting peptic peptides were trapped and desalted in a BEH C_18_ (2.1 × 5 mm, 1.7 um) VanGuard precolumn (Waters Corp., Wilmslow, UK) and separated using a Waters BEH C18 analytical column (1.0 × 100 mm, 1.7 μm) with an 8 min linear gradient of 0.1% formic acid in acetonitrile increasing from 13 to 40% at 40 μL min^–1^. To avoid peptide carryover, the pepsin column was washed two times after each run with 100 μL of pepsin wash (2 M guanidine HCl, 5% acetonitrile, 100 mM phosphate buffer, pH 2.5) and blanks ran every 3 runs. Peptide masses were measured using a Waters SELECT SERIES cIMS QTOF system. The ESI source was operated in positive ionization mode, TOF in V-Mode with capillary voltage, and sample cone of 3 kV and 40 V respectively. Different collision energy ramps were used as shown in Table 1. MS spectra (50–2000 *m*/*z*) were acquired with a 2.5 Hz scan time. On the cIMS device, a single pass was applied with a 10 ms injection, 2 ms separation, and 34 ms ejection/acquire sequence with a traveling wave (TW) static height of 22 V, ADC start delay of 12 ms and using 2 pushes per bin. For XylE experiments, an identical workflow was followed but using a 29 μM stock solution with a 10 mM sodium phosphate, 0.05% DDM pH 7.0 as the equilibration buffer and 100 mM sodium phosphate pH 2.3 as the quench buffer. Finally, for the SMO experiments, 7 μL of a 7 μM stock solution was used with 50 mM HEPES, 200 mM NaCl 0.03/0.003% DDM/CHS, pH 7.4 as equilibration buffer and 100 mM glycine, 100 mM TCEP, 4 M Urea, 0.1%DDM, pH 2.3 as quench buffer. All experiment were run minimum in triplicate (*n* ≥ 3).

**Table 1:** Average Number of Identified Peptides, Sequence Coverage, Peptide Redundancy, and PLGS Score Using Different CE Ramps for Phosphorylase B, XylE, and SMO Proteins^a^

	Phosphorylase B				XylE				SMO			
CE ramp (eV)	Peptides after curation	Coverage (%)	Redundancy	Score	Peptides after curation	Coverage (%)	Redundancy	Score	Peptides after curation	Coverage (%)	Redundancy	Score
10–50	468	90.9	5.79	7.86	348	98.2	6.60	8.04	133	73.4	2.93	7.63
10–60	419	84.8	5.51	7.86	337	98.6	6.32	8.00	118	68.3	2.89	7.61
20–45	463	93.7	5.71	7.78	199	93.7	4.15	7.57	130	72.7	3.03	7.68
20–50	396	88.1	5.13	7.82	342	98.4	6.57	8.01	131	73.8	2.95	7.66
25–45	371	83.7	5.15	7.86	330	97.0	6.65	7.97	106	59.1	2.86	7.63
25–50	267	75.2	4.06	7.66	219	93.9	4.61	7.72	121	68.0	2.94	7.63
30–45	336	89.3	4.58	7.62	202	94.1	4.50	7.67	111	66.0	2.83	7.62
30–50	282	78.4	4.43	7.59	264	95.3	5.54	7.72	79	51.0	2.41	7.57
35–45	264	78.0	4.28	7.62	200	91.7	4.43	7.58	91	56.9	2.57	7.49
35–50	25	21.9	1.71	6.97	134	84.1	3.16	7.39	54	34.2	2.09	7.31
40–45	3	4.3	1.28	6.78	179	88.3	4.08	7.49	48	34.6	1.85	7.25

a All values are the average of at least three technical replicates (*n* ≥ 3).

### Data Analysis

Raw data was analyzed using ProteinLynx Global Server software (PLGS, v3.0.3, Waters Corp.) using an updated apex3D file provided by Waters to analyze cIMS data. All PLGS parameters were kept as a default unless otherwise stated. Lock mass charge was set to *m*/*z*: 556.2771, low energy threshold set to 250 counts, elevated energy threshold set to 100 counts, minimum fragment ions set to one, primary digest reagent set to nonspecific, missed cleavages to zero, fixed modifier reagents and variable modifier reagents left blank, and false discovery rate set to 100. PLGS output files were imported into DynamX (v3.0, Waters Corp.) where more stringent peptide threshold parameters were applied. Minimum intensity set to 1000, minimum sequence length set to 4, maximum sequence length set to 25, minimum products set to 3, minimum products per amino acid set to 0.11, minimum consecutive products set to 1, minimum sum intensity for products to 472, minimum score set to 6.62, and maximum error to 5 ppm. To calculate average peptide length and score, an R script was used as detailed in the Supporting Information.

## Results and Discussion

Prior to an HDX-MS experiment, peptide sequences of peptides resulting from the proteolytic digestion of the nondeuterated protein must be confirmed. Proper peptide identification is critical and can increase the sequence coverage of the protein, resulting in additional structural information. We used phosphorylase B, XylE, and SMO proteins as model systems to optimize the CE ramp used in HDMS^E^ experiments as part of an HDX workflow in a SELECT SERIES cIMS-QTOF, to increase the number of positive identifications of peptic peptides.

### Phosphorylase B

**Figure 1:**
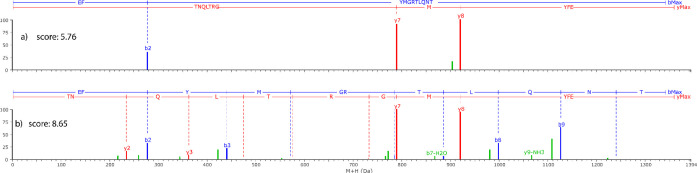
Fragment ions for PhosB peptic peptide EFYMGRTLQNT using two different collision energy ramps. (a) Fragment ions using 30–50 eV resulting in a peptide score of 5.76. (b) Fragment ions using 10–50 eV resulting in a peptide score of 8.65. Blue bars represent b fragments, red lines y fragments, and green lines b or y fragments after water or ammonia losses.

PhosB is a soluble protein commonly used to evaluate the performance of an HDX system. We started by running IMS-MS^E^ experiments with PhosB using 11 different collision energy ramps including wide ranges such as 10–60 eV and short ranges like 40–45 eV. Interestingly, the highest amount of identified PhosB peptides (2035) was observed when using an intermediate CE ramp, 20–45 eV, while the least amount of identified peptides (224) was obtained when using the shortest range in our experiment, 40–45 eV (see Table S1). Although there are clear differences in the total amount of identified peptides when changing the CE ramp, most of the identified peptides have low scores and might be false positives (data not shown), meaning that the quality of the MS^E^ data is low and the confidence of the identification is poor. To exemplify the importance of PLGS peptide score to properly identify peptides, we selected a PhosB peptic peptide (sequence: EFYMGRTLQNT) identified using multiple CE ramps. Figure 1 presents the fragment ions produced from the same peptides acquired with two collision energy ramps resulting in a score of 5.76 when using 35–50 eV and 8.65 when 10–50 eV is employed.

Although the peptide was identified with both CE ramps, when the peptide scored 5.76, only 4 product ions were identified, while when the score is 8.65 more than 15 product ions were matched giving more confidence of a positive identification. PLGS score not only considers the number of fragments identified but also their abundance and mass error among others, to provide the final number. The higher the score, the better the fragmentation of the peptide, resulting in higher certainty for the identification.

To avoid false positive identifications, we used DynamX software and applied more stringent parameters to obtain high confidence peptides only (see Experimental Section). This reduces the total amount of peptides drastically, but the certainty and quality of the identification are improved. Using this data set, a collision energy ramp of 10–50 eV gave the highest number of identified peptides with 468, and CE 20–45 eV gave the second highest amount with 463. These high number of peptides is reflected in a sequence coverage of 90.9 and 93.7% respectively with a peptide redundancy of 5.79 and 5.71 (see Table 1). All other CE ramps employed in the experiments had lower amounts of identified peptides ranging from 419 to 3 for the 10–60 and 40–45 eV ramps. As expected, the higher the number of identified peptides, the higher the redundancy. However, the difference in the average peptide score between CE ramps is not very accentuated. This is due to the stringent parameters used to filter the data, allowing only peptides with a minimum score of 6.62. On the other hand, average peptide length (see Table S1) seems to increase when CE ramps with shorter intervals (e.g., 40–45, 35–50) are used, suggesting only longer peptides are properly fragmented using those CE intervals. All in all, data demonstrate that selecting an appropriate CE ramp for the fragmentation experiments is critical for a successful peptide mapping experiment, where the sequence coverage map can change from 93.7 to 4.3% or peptide redundancy can change from 5.79 to 1.28 if not chosen appropriately.

Although Phosphorylase B gives us a glimpse of how the CE ramps change the amount of identified peptides, when working with membrane proteins, the behavior could change due to the increased complexity of the sample and the presence of membrane mimetics needed for solubilization. Consequently, we chose two proteins from different membrane protein classes, XylE and SMO, to test the influence of the CE ramps in the peptide identification.

### Membrane Proteins

XylE, a proton-coupled sugar transporter and a member of the major facilitator superfamily (MFS) that folds into 12 transmembrane domains, and Smoothened protein (SMO), a G protein-coupled receptor (GPCR) involved in the hedgehog signaling pathway containing 7 transmembrane domains, have been previously studied using HDX-MS.^29,35,36^ We used these two membrane proteins solubilized in DDM as model systems to continue testing different collision energies ramps in HDMS^E^ experiments (see Tables 1, S2, and S3).

**Figure 2:**
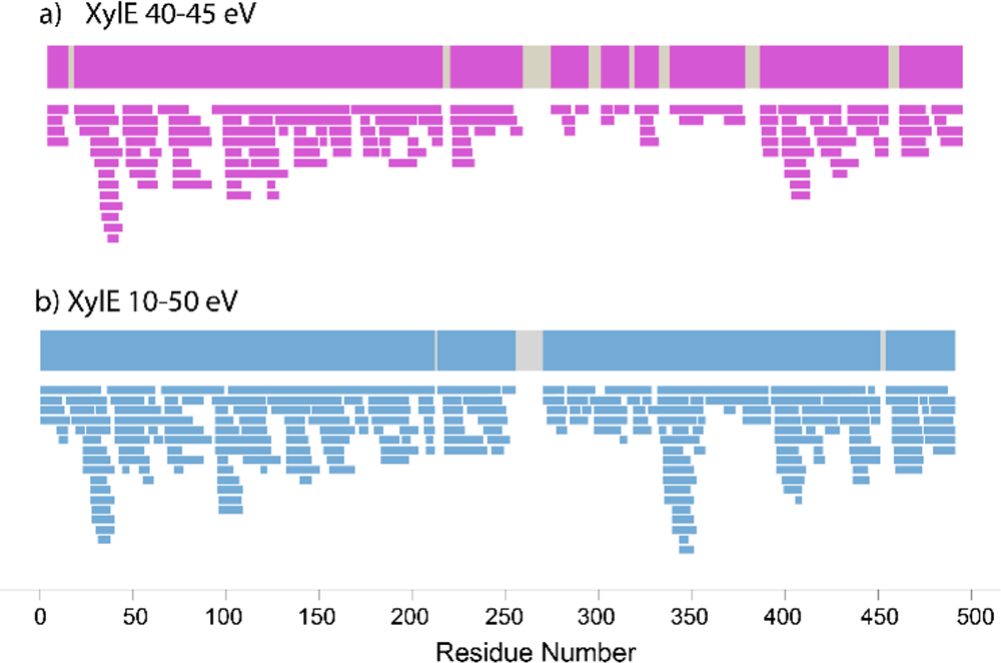
Peptide coverage map for XylE using different collision energy ramps: (a) coverage map using 40–45 eV and (b) coverage map using 10–50 eV.

Again, as with Phosphorylase B, XylE had the highest amount of identified peptides when a 10–50 eV CE ramp was used, giving 348 identified peptides, 98.2% sequence coverage, and 6.60 peptide redundancy. Closely, ramps using 10–60, 20–50, and 25–45 eV also gave a similar number of positive identifications with 337, 342, and 330 peptides. All other CE ramps used in the experiment had less than 300 positive identifications, resulting in a decrease in sequence coverage and decreased peptide redundancy. Figure 2 shows the peptide coverage map for XylE when using two different CE ramps, 40–45 and 10–50 eV with a sequence coverage of 88.3 and 98.2%, respectively. Although the difference in sequence coverage is close (∼10%), when using the 10–50 eV ramp, 169 more positive identifications are obtained, resulting in increased redundancy from 4.08 to 6.60. Increased peptide redundancy increases the resolution of the HDX observations and is argued to be even more important that sequence coverage in some cases.^37^ Data show that selecting an appropriate collision energy ramp results in increased redundancy and coverage at the same time.

CE ramps seem to not affect the number of positive identifications as much when using SMO compared with XylE or PhosB. Similar amounts of identified peptides were obtained using 10–50, 20–45, and 20–50 eV CE ramps with ∼130 peptides. CE ramps 10–60, 25–45, 25–50, and 30–45 eV showed ∼115 peptides, while all others ramps had less than 100 positive identifications. This unusual behavior suggests that efficient fragmentation of SMO peptic peptides is challenging, more likely due to inefficient digestion (e.g., longer peptides) by pepsin as has been suggested before,^38^ but also suggests that ideal CE ramps for proper peptide identification are protein and peptide specific.^39^Table S3 shows the average peptide length for the SMO identified peptides vary from 11.3 to 12.8, higher values than the ones obtained for PhosB or XylE, evidencing that indeed the peptic peptides are long due to inefficient digestion. Table 1 also shows instances where, despite a decrease in the amount of identified peptides, the sequence coverage increases. This indicates that each CE ramp has the capability to positively identify a unique set of peptic peptides.

To test this hypothesis, we pooled the identification results for SMO using multiple collision energy ramps (data not shown), resulting in 172 unique peptides with a sequence coverage of 79.9% and a redundancy of 3.59. By pooling the identification results, the number of identified peptides, sequence coverage, and redundancy dramatically increased, demonstrating that each CE ramp can indeed properly fragment a unique set of peptides.

Although the CE ramp of 10–50 eV seems to give the highest amount of positive identification for PhosB, XylE, and SMO, our results do not point to a unique CE ramp to properly fragment all peptic peptides. To the contrary, we show that CE ramp efficiency is protein specific, but more importantly, we show that different CE ramps can result in a different set of positively identified unique peptides. With that in mind, we design a simple strategy for HDX-MS users employing a Waters SELECT SERIES cIMS QTOF system to improve their peptide sequence coverage by using multiple CE ramps in their HDMS^E^ experiments. We suggest that users start their experiments using the default CE ramp (25–45 eV). In case the results are not sufficient, we recommend using alternative wider CE ramps (10–50, 10–60, and 20–50 eV) and pooling all the identification results. This process will ensure that most of the peptic peptides are properly fragmented and identified.

Finally, it is worth recalling that our experiments are optimizing the CE ramp for the proper identification of a set of peptic peptides but not optimizing the digestion efficiency of the protein itself. This is a separate process that involves the optimization of quench buffer, protease used, temperature, and protein concentration. CE ramp is not commonly optimized in an HDX workflow; however, our results demonstrate that selecting an appropriate ramp can significantly change the results of the identification.

## Conclusions

In an effort to optimize the CE ramp for HDMS^E^ experiments with membrane proteins by using a SELECT SERIES cIMS QTOF mass spectrometer as part of an HDX-MS workflow, we demonstrated the need for an appropriate CE ramp to obtain sufficient sequence coverage. CE ramp 10–50 eV gave the highest number of positive identifications in all our model proteins. But more importantly, we showed that different CE ramps can identify a unique set of peptides. We therefore recommend cIMS practitioners to use numerous CE ramps when running peptide mapping experiments and pool the results. This guarantees that all unique peptides only identified with certain CE ramps are all included in the posterior HDX analysis.

## Supporting Information Available

Additional experimental details, total peptide identifications, number of peptides after curation, sequence coverage, redundancy, average peptide length, and average peptide score for Phosphorylase B, XylE, and SMO membrane proteins when using different CE ramps (PDF)
