# Supplementary material for: MSe Collision Energy Optimization for the Analysis of Membrane Proteins Using HDX-cIMS
Source: J Am Soc Mass Spectrom. 2024 Jun 6;35(7):1383–9. doi: 10.1021/jasms.4c00093 (PMC11228973; doi:10.1021/jasms.4c00093)
Supplement: Supplementary file 1 — js4c00093_si_001.pdf [file js4c00093_si_001.pdf]

Supporting Information for:

MSe collision energy optimization for the analysis of membrane proteins using HDX-cIMS

Juan Pablo Rincon Pabon, Zulaikha Akbar, Argyris Politis*

Faculty of Biology, Medicine and Health, Division of Molecular and Cellular Function, The University of Manchester, Manchester M13 9PT, UK.

Manchester Institute of Biotechnology, University of Manchester, Princess Street, Manchester, M1 7DN, UK.

Corresponding author: argyris.politis@manchester.ac.uk

ADDITIONAL EXPERIMENTAL SECTION

Average peptide length and average peptide score for each collision energy ramp, were calculated using an R script. The script takes identification results directly from PLGS, applies the same stringent peptide thresholds as done in Dynamix software and calculates the average peptide length and average PLGS score from each CE ramp.

```
library(tidyverse) #load necessary R libraries

PLGSfile=read.csv(file.choose()) #import PLGS peptide identification results

filtered= { #applies peptide thresholds
        PLGSfile %>%
        filter(protein.Entry=="SMO1") %>%
        filter(peptide.seqLength>=4) %>% filter(peptide.seqLength<=25) %>%
        filter(peptide.MatchedProducts>=3) %>%
        filter(peptide.ConsectiveMatchedProducts>=1) %>%
        filter(peptide.score >=6.62) %>%
        filter(peptidePrecursor.deltaMhpPPM >=-5) %>% filter(peptidePrecursor.deltaMhpPPM <=5)%>%
        filter(((peptide.MatchedProducts)/(peptide.seqLength))>=0.11) %>%
        filter(peptide.MatchedProductsSumInten>=472) %>%
        filter(precursor.inten>=1000)
}

filtered=filtered[!duplicated(filtered$peptide.seq),] #removes duplicate values if any

mean(filtered$peptide.seqLength) #calculates average peptide length
mean(filtered$peptide.score) #calculates average PLGS score
```

Table S1. **Total number of identified peptides, identified peptides after curation, sequence coverage, peptide redundancy, average peptide length and average PLGS score for each replicate using different CE ramps for Phosphorylase B.**

	**Phosphorylase B**											
CE ramp (eV)	Total peptides	Average total peptides	Peptides after curation	Average peptides after curation	Sequence coverage (%)	Average sequence coverage (%)	Redundancy	Average redundancy	Peptide length	Average peptide length	Peptide score	Average peptide score
10-50	1468	1904	448	468	83.1	90.9	5.92	5.79	9.53	9.50	7.63	7.86
	1584		463		85.4		6.05		9.28		8.01	
	2124		469		95.4		5.55		9.82		7.98	
	2333		500		95.7		6.04		9.43		8.04	
	2013		460		95.0		5.41		9.45		7.66	
10-60	1543	1601	407	419	82.7	84.8	5.50	5.51	9.45	9.43	7.89	7.86
	1594		420		85.5		5.53		9.50		7.70	
	1666		429		86.1		5.51		9.35		7.99	
20-45	1571	2035	442	463	84.6	93.7	5.82	5.71	9.80	9.77	7.55	7.78
	2102		440		96.1		5.31		9.41		8.01	
	2326		496		98.1		5.95		9.96		7.79	
	2141		473		96.0		5.76		9.90		7.77	
20-50	1221	1411	377	396	84.4	88.1	5.15	5.13	9.55	9.65	7.58	7.82
	1188		379		84.1		5.16		9.73		7.89	
	1823		431		95.8		5.08		9.67		8.00	
25-45	1261	1168	376	371	81.8	83.7	5.31	5.15	9.73	9.78	7.86	7.86
	1178		381		84.9		5.23		9.84		7.90	
	1064		356		84.3		4.90		9.77		7.82	
25-50	1139	1013	344	267	81.7	75.2	4.91	4.06	9.85	10.27	7.83	7.66
	1253		349		81.1		5.00		9.81		7.88	
	647		108		62.8		2.27		11.14		7.27	
30-45	977	1538	308	336	80.3	89.3	4.66	4.58	9.96	10.08	7.51	7.62
	1784		335		93.9		4.44		10.03		7.52	
	1854		365		93.6		4.63		10.25		7.83	
30-50	1059	1019	298	282	80.4	78.4	4.52	4.43	10.10	10.31	7.74	7.59
	944		268		77.8		4.29		10.47		7.48	
	1053		281		77.0		4.47		10.35		7.53	
35-45	892	970	249	264	76.6	78.0	4.08	4.28	10.58	10.65	7.49	7.62
	1042		271		79.5		4.28		10.60		7.73	
	975		272		77.8		4.47		10.76		7.63	
35-50	458	394	47	25	41.6	21.9	1.63	1.71	12.15	12.73	7.06	6.97
	387		14		14.0		1.62		13.64		7.00	
	337		13		10.2		1.87		12.38		6.87	
40-45	268	224	3	3	4.0	4.3	1.47	1.28	16.67	15.81	6.79	6.78
	227		4		5.3		1.36		15.25		6.73	
	177		2		3.7		1.00		15.50		6.82	

***Table S2.* Total number of identified peptides, identified peptides after curation, sequence coverage, peptide redundancy, average peptide length and average PLGS score for each replicate using different CE ramps for XylE.**

	XylE											
CE ramp (eV)	Total peptides	Average total peptides	Peptides after curation	Average peptides after curation	Sequence coverage (%)	Average sequence coverage (%)	Redundancy	Average redundancy	Peptide length	Average peptide length	Peptide score	Average peptide score
10-50	834	831	337	348	99.2	98.2	6.37	6.60	9.21	9.14	7.98	8.04
	834		352		96.1		6.80		9.12		8.03	
	826		355		99.2		6.64		9.10		8.11	
10-60	750	803	319	337	96.7	98.6	6.01	6.32	8.95	9.08	7.98	8.00
	800		341		99.8		6.37		9.16		7.97	
	859		351		99.2		6.59		9.14		8.05	
20-45	604	561	271	199	94.9	93.7	5.21	4.15	8.96	9.74	7.65	7.57
	509		210		93.3		4.14		9.03		7.49	
	468		175		90.2		3.38		8.56		7.51	
	639		166		95.9		3.96		11.22		7.64	
	585		172		94.1		4.06		10.92		7.56	
20-50	766	782	332	342	99.8	98.4	6.29	6.57	9.29	9.28	8.02	8.01
	809		337		96.1		6.55		9.17		8.04	
	770		356		99.2		6.86		9.39		7.99	
25-45	787	791	325	330	98.6	97.0	6.48	6.65	9.65	9.59	7.79	7.97
	763		330		94.7		6.76		9.52		8.07	
	823		336		97.8		6.72		9.60		8.05	
25-50	776	591	318	219	98.8	93.9	6.25	4.61	9.52	9.87	8.05	7.72
	570		254		93.1		5.18		9.33		7.77	
	451		172		89.2		3.58		9.12		7.57	
	605		189		95.3		4.35		10.77		7.63	
	551		160		93.1		3.71		10.61		7.58	
30-45	680	599	276	202	95.7	94.1	5.74	4.50	9.78	10.51	7.84	7.67
	705		277		96.3		5.72		9.77		7.93	
	502		146		92.9		3.51		10.96		7.55	
	572		158		91.9		3.80		10.86		7.53	
	534		153		93.5		3.72		11.17		7.48	
30-50	706	724	259	264	93.3	95.3	5.52	5.54	9.76	9.81	7.73	7.72
	743		276		96.3		5.74		9.83		7.74	
	722		258		96.3		5.37		9.83		7.71	
35-45	647	584	229	200	96.6	91.7	4.96	4.43	9.73	9.91	7.63	7.58
	672		246		94.5		5.28		9.95		7.72	
	477		158		87.0		3.42		9.24		7.42	
	538		166		88.8		4.07		10.70		7.54	
35-50	361	500	104	134	80.9	84.1	2.37	3.16	9.05	9.90	7.25	7.39
	569		192		89.6		4.26		9.77		7.52	

	570		105		81.9		2.84		10.88		7.39	
40-45	619	580	193	179	88.8	88.3	4.35	4.08	9.82	9.90	7.53	7.49
	659		197		89.8		4.40		9.84		7.58	
	463		147		86.4		3.48		10.03		7.37	

Table S3. **Total number of identified peptides, identified peptides after curation, sequence coverage, peptide redundancy, average peptide length and average PLGS score for each replicate using different CE ramps for SMO protein.**

	SMO											
CE ramp (eV)	Total peptides	Average total peptides	Peptides after curation	Average peptides after curation	Sequence coverage (%)	Average sequence coverage (%)	Redundancy	Average redundancy	Peptide length	Average peptide length	Peptide score	Average peptide score
10-50	1842	2419	121	133	68.8	73.4	2.78	2.93	11.16	11.41	7.60	7.63
	2066		125		69.8		2.79		11.00		7.61	
	2110		132		74.5		2.81		11.18		7.67	
	3659		153		80.5		3.32		12.32		7.64	
10-60	2472	2030	127	118	72.9	68.3	2.90	2.89	11.80	11.81	7.61	7.61
	2382		134		77.1		3.04		12.39		7.56	
	1236		94		54.8		2.73		11.24		7.66	
20-45	2012	2121	128	130	72.0	72.7	2.96	3.03	11.75	11.92	7.69	7.68
	2097		129		77.1		2.85		12.01		7.68	
	2255		133		69.1		3.27		11.99		7.67	
20-50	2226	2188	137	131	76.6	73.8	3.04	2.95	12.05	11.77	7.65	7.66
	2219		124		71.2		2.81		11.39		7.70	
	2238		135		73.7		3.05		11.77		7.66	
	2070		127		73.7		2.90		11.86		7.63	
25-45	1281	1554	88	106	54.0	59.1	2.60	2.86	11.27	11.34	7.67	7.63
	1317		104		58.1		2.85		11.24		7.63	
	2063		125		65.2		3.12		11.50		7.57	
25-50	1957	2010	116	121	63.2	68.0	2.91	2.94	11.22	11.66	7.67	7.63
	1953		128		72.7		2.99		11.98		7.57	
	2123		123		66.6		3.04		11.63		7.62	
	2007		117		69.3		2.82		11.81		7.67	
30-45	2074	1794	116	111	62.0	66.0	3.07	2.83	11.62	11.82	7.69	7.62
	2065		123		78.0		2.70		12.11		7.60	
	1243		95		58.1		2.72		11.73		7.58	
30-50	1277	1272	63	79	47.5	51.0	2.08	2.41	11.08	11.07	7.59	7.57
	1284		84		52.3		2.52		11.06		7.57	
	1256		89		53.3		2.62		11.08		7.56	
35-45	1348	1298	89	91	59.6	56.9	2.46	2.57	11.62	11.40	7.53	7.49
	1336		93		57.2		2.63		11.50		7.49	
	1210		90		54.0		2.62		11.08		7.46	

35-50	1191	1389	78	54	51.6	34.2	2.39	2.09	11.15	12.78	7.52	7.31
	1291		82		46.6		2.89		11.67		7.55	
	1686		2		4.4		1.00		15.50		6.85	
40-45	1759	1337	4	48	9.5	34.6	1.00	1.85	16.75	12.83	6.72	7.25
	1012		62		41.1		2.37		11.06		7.51	
	1241		77		53.3		2.19		10.69		7.53	
